# Effects of Dopamine on the Immature Neurons of the Adult Rat Piriform Cortex

**DOI:** 10.3389/fnins.2020.574234

**Published:** 2020-10-06

**Authors:** Simona Coviello, Yaiza Gramuntell, Esther Castillo-Gomez, Juan Nacher

**Affiliations:** ^1^Neurobiology Unit, Program in Neurosciences and Institute of Biotechnology and Biomedicine (BIOTECMED), Universitat de València, Burjassot, Spain; ^2^Department of Medicine, School of Medical Sciences, Universitat Jaume I, Castellón de la Plana, Spain; ^3^Spanish National Network for Research in Mental Health (CIBERSAM), Madrid, Spain; ^4^Fundación Investigación Hospital Clínico de Valencia, INCLIVA, Valencia, Spain

**Keywords:** piriform cortex, dopamine, dopamine D2 receptor, plasticity, PSA-NCAM

## Abstract

The layer II of the adult piriform cortex (PCX) contains a numerous population of immature neurons. Interestingly, in both mice and rats, most, if not all, these cells have an embryonic origin. Moreover, recent studies from our laboratory have shown that they progressively mature into typical excitatory neurons of the PCX layer II. Therefore, the adult PCX is considered a “non-canonical” neurogenic niche. These immature neurons express the polysialylated form of the neural cell adhesion molecule (PSA-NCAM), a molecule critical for different neurodevelopmental processes. Dopamine (DA) is a relevant neurotransmitter in the adult CNS, which also plays important roles in neural development and adult plasticity, including the regulation of PSA-NCAM expression. In order to evaluate the hypothetical effects of pharmacological modulation of dopaminergic neurotransmission on the differentiation of immature neurons of the adult PCX, we studied dopamine D2 receptor (D2r) expression in this region and the relationship between dopaminergic fibers and immature neurons (defined by PSA-NCAM expression). In addition, we analyzed the density of immature neurons after chronic treatments with an antagonist and an agonist of D2r: haloperidol and PPHT, respectively. Many dopaminergic fibers were observed in close apposition to PSA-NCAM-expressing neurons, which also coexpressed D2r. Chronic treatment with haloperidol significantly increased the number of PSA-NCAM immunoreactive cells, while PPHT treatment decreased it. These results indicate a prominent role of dopamine, through D2r and PSA-NCAM, on the regulation of the final steps of development of immature neurons in the adult PCX.

## Introduction

In the last two decades, many studies have been conducted to characterize a population of immature neurons, which is densely distributed in the layer II of the adult rodent piriform cortex (PCX). Most, if not all, of these immature neurons are postmitotic cells generated during embryonic development (Castillo-Gómez et al., 2008; Rubio et al., 2016), which progressively mature into typical excitatory neurons of the PCX (Rotheneichner et al., 2018; Benedetti et al., 2019). These immature neurons transiently express the polysialylated form of the neural cell adhesion molecule (PSA-NCAM) (Seki and Arai, 1991; Bonfanti et al., 1992), a plasticity molecule involved in different neurodevelopmental processes, such as neuronal migration, neurite extension, or synaptogenesis (Rutishauser, 2008; Hildebrandt and Dityatev, 2013), which may also act as an insulating agent on these cells, preventing or limiting the formation of synaptic contacts (Gómez-Climent et al., 2008). PSA-NCAM-expressing cells in the PCX of rodents can be divided into two morphological categories, although intermediate forms are very common: (a) tangled (type I) cells, which had a very small somata, lack axons, and have few short dendrites with intricate trajectories and (b) complex (type II) cells, which show typical dendritic branches and resemble common excitatory neurons in the PCX layer II (see Bonfanti and Nacher, 2012 for review). PSA-NCAM-expressing cells also coexpress markers of immature neurons such as doublecortin or TUC4 and lack or have very low expression of mature neuronal markers, such as calcium calmodulin kinase IIα (CAMKII) or NeuN (Gómez-Climent et al., 2008; Rubio et al., 2016). However, these immature neurons express transcription factors exclusive of excitatory neurons and lack those expressed by interneurons (Gómez-Climent et al., 2008; Bonfanti and Nacher, 2012; Rubio et al., 2016). The function of these immature neurons in the PCX is still a mystery, but some studies have indicated that they are affected by chronic stress and chronic corticosterone treatment (Nacher et al., 2004), as well as by bulbectomy (Gómez-Climent et al., 2011), which are considered animal models of major depression (Planchez et al., 2019). Moreover, this population of neurons is also affected by antidepressant treatment (Sairanen et al., 2007). Together, these results suggest the involvement of these neurons in the response to adverse experiences and depressive-like behavior. This is also supported by recent work in Dr. Vaidya’s laboratory, which demonstrated the contribution of the monoamine norepinephrine, a neurotransmitter involved in the response to stress and the therapeutics of major depression, in the regulation of the number of PSA-NCAM-expressing cells in the PCX of adult rats (Vadodaria et al., 2017). Interestingly, previous studies from our laboratory have demonstrated that PSA-NCAM levels in the cerebral cortex are regulated by another monoamine, dopamine, acting through dopamine D2 receptor (D2r) (Castillo-Gómez et al., 2008, 2016), although these studies were focused on the expression of PSA-NCAM in prefrontocortical interneurons. The role of PSA-NCAM in response to monoamines and, in general, to stress and other aversive experiences in the adult brain must be related to its capacity to induce plastic phenomena, such as synaptic and dendritic remodeling, and also to its insulating properties, which could limit transiently this plasticity (Nacher et al., 2013). The monoamines influence the proliferation of neuronal stem cells (NSCs) within the two neurogenic niches of the adult brain: the subventricular zone (SVZ) and the subgranular zone (SGZ) of the hippocampal dentate gyrus (Apple et al., 2017). In particular, dopamine appears to control neurogenesis in both SVZ and SGZ, as indicated by its experimental manipulation (Höglinger et al., 2004). In a 6-OHDA rat model of Parkinson’s disease, dopamine agonists induce a significant increase in SVZ proliferation and seem to promote neuronal differentiation in the olfactory bulb of 6-OHDA lesioned animals (O’Keeffe et al., 2009; Winner et al., 2009). These actions of dopamine are also evident during neurodevelopment, when it plays important roles in the regulation of different stages of neural development, including cell proliferation, migration, and differentiation (Money and Stanwood, 2013). Cells in the PCX receive inputs from dopaminergic neurons and express dopamine receptors, especially D2 (Boyson et al., 1986). However, the expression of these receptors specifically by the immature neurons in layer II and the relationship between these cells and dopaminergic terminals have not been explored yet. Therefore, considering that dopamine plays a key role in the response to stress and the etiopathology and treatment of major depression (Belujon and Grace, 2015, 2017), and that it is a potent regulator of neuronal development via its receptors (Todd, 1992; Schmidt et al., 1996; Sung et al., 2006), it would be interesting to investigate the influence of the pharmacological modulation of dopaminergic neurotransmission on the population of immature neurons in the PCX layer II. Consequently, to evaluate the hypothetical effects of D2r antagonists and agonists on the differentiation of immature neurons in the PCX layer II, we first studied the expression of D2r on these cells and their relationship with dopaminergic axons. Next, we explored whether the experimental manipulation of dopaminergic signaling via D2r antagonists and agonists may alter the rate of differentiation of the immature neurons in the PCX by quantifying changes in the density of PSA-NCAM-expressing cells in layer II.

## Materials and Methods

### Animals

Twenty-four male Sprague–Dawley rats (3 months old, 300 ± 15 g, Harlan Iberica) were used in this study. Two rats were used to study the expression of dopamine D2 receptors (D2r) in PSA-NCAM immunoreactive cells and the spatial relationship between these cells and fibers immunopositive for tyrosine hydroxylase (TH) and the dopamine transporter (DAT). Twenty-two rats were used for experimental manipulations of the dopamine D2 receptors: 10 rats were used for the D2r antagonist (haloperidol) experiment, and 12 rats were used for the D2r agonist [2-(N-Phenethyl-N-propyl) amino-5-hydroxytetralin hydrochloride (PPHT)] experiment. The animals were housed in groups in a temperature and humidity-controlled environment and were maintained on a 12-h light/dark cycle with free access to food and water. Rats were allowed to habituate in our facilities at least 1 week prior to the start of the experiments. All animal experimentations were performed in accordance with the Directive 2010/63/EU of the European Parliament and of the Council of September 22, 2010 on the protection of animals used for scientific purposes and was approved by the Committee on Bioethics of the University of Valencia. Every effort was made to minimize the number of animals used and their suffering.

### Chronic Treatment With Haloperidol

Ten rats were divided randomly into two groups: control (vehicle-treated; *n* = 5) and haloperidol treated (0.5 mg/kg/day, *n* = 5). The injections were administered i.p. once daily for 26 consecutive days (MacDonald et al., 2005; Castillo-Gómez et al., 2008). Haloperidol (Sigma-Aldrich) was dissolved in 0.1 N acetic acid, pH 5–6. Animals were perfused transcardially 24 h after the final injection, and their brains were processed as described below for PSA-NCAM immunohistochemistry.

### Chronic Treatment With PPHT

Rats were randomly separated into two groups and administered intraperitoneally with either the selective D2r agonist 2-(N-Phenethyl-N-propyl) amino-5-hydroxytetralin hydrochloride (PPHT, *n* = 6, 1.5 mg/kg/day in 0.9% NaCl; Sigma-Aldrich) or saline (*n* = 6) once daily for 7 consecutive days (Sugahara and Shiraishi, 1998; Castillo-Gómez et al., 2008, Castillo-Gómez et al., 2011). The animals were sacrificed 24 h after the final injection. Their brains were processed, and sections were analyzed using immunohistochemistry for PSA-NCAM as described below.

### Histology

All rats (*n* = 24) were perfused transcardially under deep chloral hydrate anesthesia, first with saline and then with 4% paraformaldehyde in sodium phosphate buffer 0.1 M, pH 7.4. Thirty minutes after perfusion, the brains were extracted and cryoprotected for 48 h with 30% sucrose in 0.1 M phosphate buffer (PB). Coronal sections (50 μm) were obtained from frozen brains (−30°C) with a freezing–sliding microtome (Leica SM2000R), collected in 10 subseries and stored at −20°C in 30% glycerol, 30% ethylene glycol in PB until used. One subseries of sections was stained with toluidine blue and used to better delimitate the boundaries of the PCX and its different layers.

### Immunohistochemistry for Conventional Light Microscopy

Tissue was processed “free-floating” for immunohistochemistry as follows: Briefly, sections were incubated for 1 min in an antigen-unmasking solution (0.01 M citrate buffer, pH 6) at 100°C. After cooling down the sections to room temperature, they were incubated with 10% methanol, 3% H_2_O_2_ in phosphate-buffered saline (PBS) for 10 min to block endogenous peroxidase activity. After this, sections were treated for 1 h with 10% normal donkey serum (NDS) (Jackson ImmunoResearch Laboratories, West Grove, PA, United States) in PBS with 0.2% Triton-X100 (Sigma-Aldrich, St. Louis, MO, United States) and were incubated overnight at room temperature in monoclonal mouse IgM anti-polysialic acid-NCAM antibody (1:1,400; MAB5324, Sigma-Aldrich). After washing, sections were incubated for 2 h with donkey anti-mouse IgM biotinylated antibody (1:250; Jackson ImmunoResearch Laboratories), followed by an avidin–biotin–peroxidase complex (ABC; Vector Laboratories, Peterborough, United Kingdom) for 1 h in PBS. Color development was achieved by incubating with 3,3′diaminobenzidine tetrahydrochloride (DAB; Sigma-Aldrich) and H_2_O_2_ for 4 min. PBS containing 0.2% Triton-X100 and 5% NDS was used for primary and secondary antibodies dilution. All sections passed through all procedures simultaneously in order to minimize any difference from immunohistochemical staining itself.

The anti-PSA-NCAM antibody was generated against rat embryonic spinal cord membranes. The specificity of immunostaining in our tissue was tested in four different ways, as described before (Gómez-Climent et al., 2008): (a) three different anti-PSA antibodies were used, and they rendered exactly the same pattern of immunostaining, (b) pretreatment of the antibody with a-2,8-linked sialic polymer (colominic acid, Sigma) overnight, or the primary antibody omission during the immunohistochemistry prevented all the labeling in the piriform cortex layer II, (c) PSA immunohistochemistry of sections from animals injected intracerebrally with EndoN resulted in a complete absence of PSA immunostaining in the area affected by the injection, and (d) immunostaining was absent from adult CNS tissue of St8SiaII/St8SiaIV double knockout mice.

### Immunohistochemistry for Confocal Microscopy

In general, sections were processed as described above but omitting the endogenous peroxidase block. The first day, all sections were incubated overnight at room temperature with the following primary antibody cocktails: (a) monoclonal mouse IgM anti-PSA-NCAM (1:1,400; MAB5324, Sigma-Aldrich) and polyclonal rabbit anti-human dopamine receptor (D2r) (1:200; AB5084P, Sigma-Aldrich) or (b) monoclonal mouse IgM Anti-PSA-NCAM, monoclonal rat IgG anti-dopamine transporter (DAT) (1:200; MAB369, Sigma-Aldrich), and monoclonal mouse IgG_1_ anti-tyrosine hydroxylase (TH) (1:200; MAB318, Sigma-Aldrich). Forty-eight hours later, sections were washed and incubated for 2 h with the following cocktail of fluorescent secondary antibodies: goat anti-mouse IgM conjugated with Alexa 555 (1:200; Molecular Probes, Eugene, OR, United States), donkey anti-rabbit IgG conjugated with Alexa 488 (1:200; Invitrogen), goat anti-rat IgG (cross-adsorbed) conjugated with Alexa 488 (1:200; Molecular Probes, Eugene, OR, United States) and goat anti mouse IgG_1_ conjugated with Alexa 647 (1:200; Life Technologies). All sections were mounted on slides and coverslipped using DakoCytomation fluorescent mounting medium (Dako). The omission of the primary antibodies during the immunohistochemical protocols resulted in total absence of immunostaining.

The D2r antibody was raised against a 28-amino acid fragment (284–311) within the cytoplasmic third loop (aa 284–311) of human dopamine D2r (Hersch et al., 1995). The immunogen peptide has no significant homology with other subtypes of dopamine receptors (D1, D3–D5), and the antiserum recognizes both short and long forms of D2r (Boundy et al., 1993). The specificity of the antiserum for the immunogen peptide was shown in rats by the absence of immunoreactivity in controls of preimmune antiserum and immunogen peptide adsorption (Boundy et al., 1993).

The dopamine transporter (DAT) antibody used in our study corresponds to the DAT/Nt antibody generated against human DAT N-terminus amino acids 1–66. The specificity of this antibody has been demonstrated by immunoblot analysis, immunoprecipitation, preadsorption followed by immunoblotting and immunohistochemistry, and several immunohistochemical assays, and it has been validated for the detection of dopamine transporter in rats (Wersinger et al., 2004; Bianco et al., 2008; García-Cabezas et al., 2009; Pandolfo et al., 2013).

The mouse monoclonal anti-tyrosine hydroxylase antibody was raised against purified TH from the PC 12 pheochromocytoma cell line; this antibody recognizes recombinant TH by Western blot analysis (Wolf and Kapatos, 1989). The anti-TH antibody labels a single protein band in the molecular weight range of 56–60 kDA on Western blots prepared from VTA protein homogenates (Shepard et al., 2006). In addition, this anti-TH antibody was used to demonstrate that midbrain unilateral lesion with 6-hydroxydopamine (6-OHDA) results in lack of TH immunolabeling in the lesioned site, whereas it is preserved in the contralateral unlesioned midrain (Sarabi et al., 2001). The clone LNC1 has been validated for use in immunohistochemistry with more than 700 product citations (manufacturer’s information).

### Confocal Analysis and Quantification

The sections were observed under a confocal microscope (Leica TCS-SPE). Z-series of optical sections (0.5 μm apart) of the whole thickness of the histological sections were obtained using sequential scanning mode. These stacks were processed with FIJI/ImageJ software (Schindelin et al., 2012). Fifty PSA-NCAM immunoreactive cells were randomly selected from all the sections containing PCX (10 sections between Bregma -1.40 to -3.80 mm (Herman and Watson, 1987) to analyze their spatial relationship with TH and DAT immunoreactive fibers. To obtain the percentages describing their colocalization with D2r, the quantification was performed blindly by first evaluating PSA-NCAM neurons in individual detection channels to assess the distribution of D2r population, followed by merging the single images to identify and count doubly labeled neurons. In addition, 25 TH fibers were also selected from the same sections to analyze their colocalization with DAT.

### Quantification of the Density of PSA-NCAM Expressing Cells

In order to study the density of PSA-NCAM-expressing cells in the PCX layer II (haloperidol and PPHT experiments), two sections per animal were selected from the following coordinates: bregma -1.40 mm and bregma -3.60 mm (Herman and Watson, 1987). Cell counting was performed manually, using an Olympus CX41 microscope, and results were expressed as density of immunopositive cells per mm^2^.

### Statistical Analyses

After checking the normality and homoscedasticity of the data, we used unpaired Student’s *t*-tests to compare control and experimental groups. Analysis was performed using the statistical package SPSS v22.0 (IBM). In every test, α was set to 0.05, and the animal was considered as the “n.”

## Results

### Relationship Between PSA-NCAM Immunoreactive Neurons and Dopaminergic Fibers in the Rat Piriform Cortex Layer II

**FIGURE 1:**
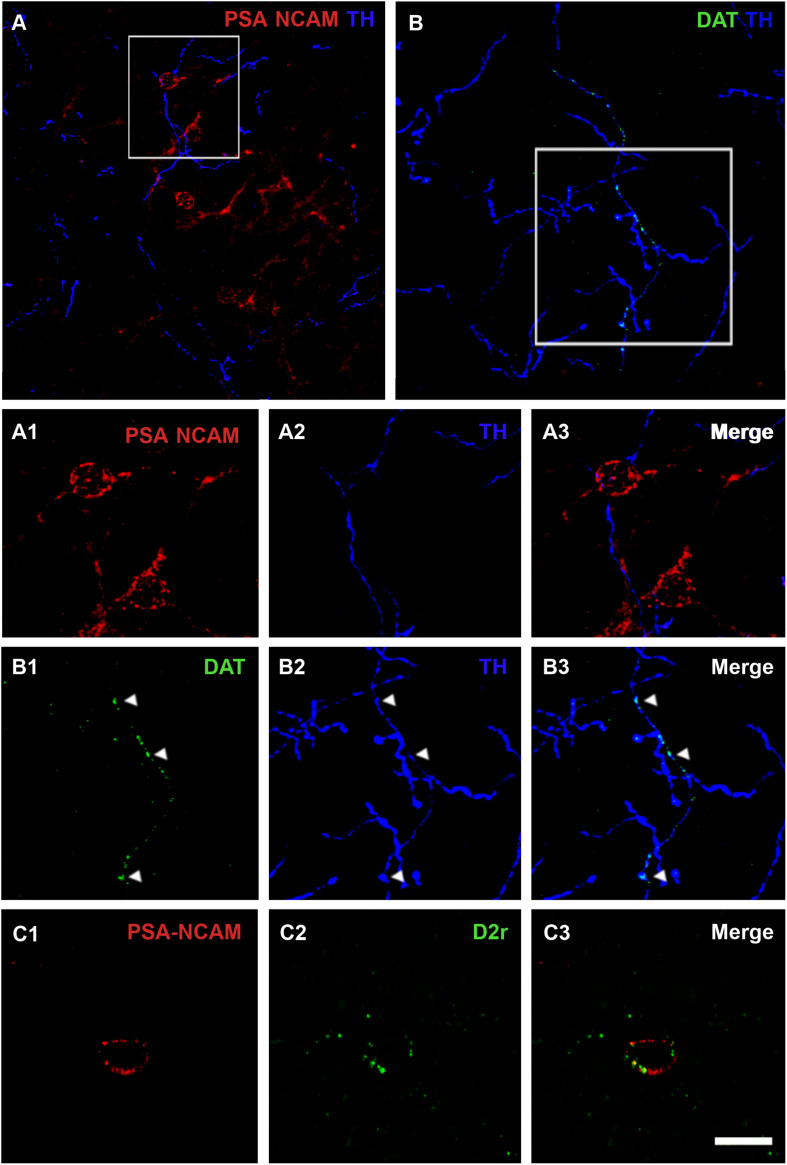
Confocal microscopic analysis of PSA-NCAM immunoreactive neurons and dopaminergic fibers in the piriform cortex layer II. **(A)** Double PSA-NCAM/TH immunohistochemistry. Note the TH immunoreactive fibers surrounding and contacting PSA-NCAM-expressing cells. In **(A1–A3)**, a detailed view of the magnification of the squared area in **(A)** can be observed. **(B)** Double DAT/TH immunohistochemistry. Observe the expression of DAT on TH fibers, squared area in **(B)**, and arrowheads in **(B1–B3)** images. **(C1)** Neuron expressing PSA-NCAM in the piriform cortex layer II. In **(C1–C3)** note the presence of D2r expression on PSA-NCAM-expressing cells. All the images in this figure are 2D projections of 25 consecutive confocal planes located 0.5 μm apart. Scale bars: 50 μm for **(A)**; 30 μm for **(B)**; 10 μm for **(C)**, and 15 μm for **(A1–A3,B1–B3)**. PSA-NCAM, polysialylated form of the neural cell adhesion molecule; TH, tyrosine hydroxylase; DAT, dopamine transporter; D2r, dopamine D2 receptor.

We analyzed whether TH immunoreactive fibers were closely apposed to PSA-NCAM immunoreactive neurons in the rat PCX layer II, using double immunostaining with antibodies against these two markers. Fibers containing TH were sparse in the three-layered PCX, but many of them were observed in close apposition to PSA-NCAM-expressing neurons in layer II (Figures 1A,A1– 3). Since TH can also be found in noradrenergic axons, we analyzed the coexpression of TH and DAT in fibers closely apposed to PSA-NCAM immunoreactive neurons, confirming their dopaminergic phenotype (Figures 1B,B1–3).

### Dopamine D2 Receptor Expression in PSA-NCAM Immunoreactive Neurons

Using double immunohistochemistry for PSA-NCAM and D2r, we also analyzed whether PSA-NCAM immunoreactive neurons in the rat PCX layer II coexpressed this receptor. Interestingly, we found that 55% of these cells coexpressed D2r. The detailed analysis of its expression showed that D2r immunoreactivity was predominantly localized in the periphery of the somata, in the same compartment where PSA-NCAM immunoreactivity can be found (Figures 1C1–3).

### Effects of Chronic Treatments With D2 Receptor Antagonists and Agonists

We next examined whether the pharmacological modulation of dopaminergic neurotransmission, using D2r antagonists and agonists, influenced the density of immature neurons expressing PSA-NCAM within the rat PCX layer II.

#### Density of PSA-NCAM Immunoreactive Cells After Chronic Haloperidol Treatment

**FIGURE 2:**
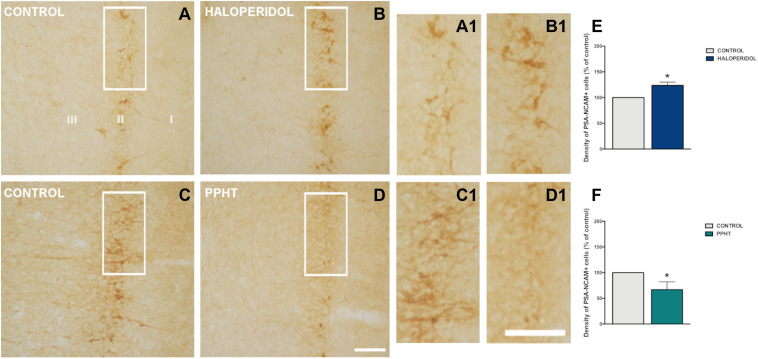
PSA-NCAM-expressing cells in the rat piriform cortex. Microphotographs showing PSA-NCAM immunostaining in control **(A,C)** and after chronic treatment with haloperidol **(B)** and PPHT **(D)**. **(A1–D1)** are higher magnifications of the squared areas in **(A–D)**. **(E,F)** Graphs showing changes in the density of PSA-NCAM-expressing cells in the piriform cortex layer II. Asterisks indicate statistically significant differences from the control group after unpaired Student’s *t*-test; *p* < 0.05 (*). Scale bars: 200 μm for **(A–D)**; 100 μm for **(A1–D1)**. PSA-NCAM, polysialylated form of the neural cell adhesion molecule; PPHT, 2-(N-phenethyl-N-propyl) amino-5-hydroxytetralin hydrochloride.

Twenty-six days of chronic haloperidol treatment induced an increase in the density of PSA-NCAM-expressing cells in the rat PCX layer II (Figures 2A,B). This increase was statistically significant (*p* = 0.028) (Figure 2E).

#### Density of PSA-NCAM Immunoreactive Cells After Chronic PPHT Treatment

Seven days of chronic PPHT treatment produced a decrease in the density of PSA-NCAM-expressing cells in the rat PCX layer II (Figures 2C,D). This reduction was statistically significant (*p* = 0.015) (Figure 2F).

## Discussion

The present study describes the relationship of dopaminergic neurotransmission with the population of immature neurons in the PCX layer II of adult rats. We have identified this population of cells by the expression of PSA-NCAM, a plasticity-related molecule, which is strongly expressed in their somata and dendrites (Seki and Arai, 1991; Bonfanti, 2006). The distribution and morphology of these cells are identical to those described previously by us and others in rats (Bonfanti et al., 1992; Nacher et al., 2002). Most of the immature neurons in the adult rat PCX layer II have been generated during embryonic development (Gómez-Climent et al., 2008) and progressively mature into functional excitatory neurons in this layer (Rotheneichner et al., 2018; Benedetti et al., 2019), although the factors triggering or modulating these final steps of differentiation are still far from being understood.

We have decided to study the influence of the pharmacological modulation of dopaminergic neurotransmission on this immature neuronal population following three lines of evidence: (1) this monoamine regulates the expression of PSA-NCAM in the adult cerebral cortex (Castillo-Gómez et al., 2008), (2) another monoamine, norepinephrine, affects this immature neuronal population (Vadodaria et al., 2017), and (3) dopamine regulates neuronal differentiation during development (Money and Stanwood, 2013).

We have described the presence of fibers expressing TH/DAT in the close vicinity of PSA-NCAM-expressing somata. In accordance with previous studies in the PCX (Plantjé et al., 1987; Datiche and Cattarelli, 1996), we have observed that several immunoreactive fibers coexpressing TH and DAT, and consequently dopaminergic, could be found apposed to PSA-NCAM-expressing somata. It is unlikely, however, that these fibers establish synapses with the immature neurons because the presence of PSA-NCAM on their membranes prevents this type of contacts. There is evidence that only the cells in the most advanced stages of maturation show dendritic spines, in which some scarce synaptic contacts can be found (Gómez-Climent et al., 2008). However, monoamines, including dopamine, act in great part by volume transmission (Vizi et al., 2010), a type of paracrine signaling and, thus, could exert their effect if receptors were present in the target neurons. In any case, further analyses using electron microscopy combined with immunohistochemistry would be necessary to unequivocally describe the relationship between dopaminergic fibers and the immature neurons.

Since previous experiments in our laboratory have demonstrated that the expression of PSA-NCAM in the prefrontal cortex can be modulated by dopamine through the pharmacological manipulation of D2r (Castillo-Gómez et al., 2008), we have studied their expression in the immature neurons in the PCX and confirmed their presence. This is consistent with previous data describing the expression of D2r in this cortical region (Meador-Woodruff et al., 1989). Although the cellular pattern of D2r expression in the PCX has not been explored yet, there is evidence of the presence of these receptors in both inhibitory and excitatory neurons in other cortical regions (Santana et al., 2009). Consequently, some of the D2r-expressing cells in the PCX may be the immature neurons in layer II, which display an excitatory phenotype and differentiate into typical excitatory neurons of this region (Gómez-Climent et al., 2008; Rubio et al., 2016; Rotheneichner et al., 2018). It is important to note that we have only detected D2r in half of the PSA-NCAM-positive cells, which raises questions on whether the expression of these receptors is restricted to certain phases of the development of these neurons. Since the presence of D1r has also been described in the PCX and in cortical excitatory cells, future studies should also study their putative expression in the immature neurons of the PCX and the influence of their specific manipulation on these cells.

Our study also shows that the pharmacological manipulation of D2r with specific antagonists and agonists produces opposite effects on the population of immature neurons in the PCX. We think that the decrease in the number of cells expressing PSA-NCAM induced by the D2r agonist PPHT may represent an increase in the rate of differentiation of these cells, which progressively lose PSA-NCAM expression as they reach their maturity (Rotheneichner et al., 2018). In fact, PSA-NCAM appears to have an insulating role in this immature neuronal population, limiting the formation of synaptic contacts (Gomez-Climent et al., 2011). On the contrary, the increase in the number of PSA-NCAM-positive cells observed after treatment with haloperidol (a D2r antagonist) may be the consequence of a decrease in the rate of differentiation of these neurons. The number of immature neurons in the PCX decreases continuously as life progresses (Varea et al., 2009; Rotheneichner et al., 2018). Consequently, if the differentiation is slowed by haloperidol, at the end of the treatment, we will detect more PSA-NCAM-expressing cells in the animals receiving this drug than in the controls, in which a larger cell population will have reached the PSA-NCAM-negative mature stage. However, further analyses with markers of mature neurons, such as NeuN or CAMKII, and the use of transgenic animals similar to those used in the Rotheneichner et al. (2018) study should be performed to demonstrate clearly this hypothesis. The effects of the pharmacological manipulation of D2r on PSA-NCAM expression have been already studied in the adult medial prefrontal cortex (mPFC) (Castillo-Gómez et al., 2008, Castillo-Gómez et al., 2011). However, the effects of these manipulations produce opposite effects to those detected in the PCX layer II. It has to be noted, however, that in the mPFC, PSA-NCAM is exclusively expressed by mature inhibitory neurons (Varea et al., 2005) and that the role of this molecule in these interneurons may be different than in the PCX immature neurons. In fact, the regulation of PSA-NCAM expression should also be different because the polysialyltransferase St8SiaII is responsible for the addition of PSA to NCAM in the immature neurons of the PCX, while St8SiaIV plays this role in mature interneurons (Nacher et al., 2010). Ongoing studies in our laboratory are exploring the effects of PSA depletion on the maturation of PCX layer II cells.

Our results on the impact of the pharmacological modulation of D2r are particularly interesting because there are different lines of evidence suggesting the involvement of the immature neurons in the adult cortical layer II in psychiatric disorders and their treatment, especially in major depression. Although in rodents this cell population is restricted mainly to the PCX, they have a wider distribution in gyrencephalic animals (Varea et al., 2011; Piumatti et al., 2017), including humans (Ní Dhúill et al., 1999; Cai et al., 2009), covering most neocortical regions. Interestingly, similar cells have also been found in the human amygdala, a region critically involved in mood disorders (Martí-Mengual et al., 2013; Sorrells et al., 2019). Previous reports from our laboratory have shown that two animal models of major depression, chronic stress, and chronic corticosterone treatment have a dramatic impact on the number of these immature neurons (Nacher et al., 2004). Olfactory bulbectomy, another accepted model of major depression, induces the differentiation of these cells (Gómez-Climent et al., 2011). Moreover, chronic treatment with the antidepressant imipramine increases PSA-NCAM expression in the PCX layer II of adult rats (Sairanen et al., 2007). These results are also interesting because imipramine, besides its action as a 5HT and norepinephrine reuptake inhibitor, also blocks D2 receptors (Smiałowski, 1991). This is consistent with the effects that we observe with haloperidol in the present study. In addition, chronic treatment with the antidepressant fluoxetine, a 5HT reuptake blocker, also induces an increase in PSA-NCAM expression in the rat entorhinal cortex layer II, a region where some PSA-NCAM + immature neurons can also be detected (Varea et al., 2007). Finally, the modulation of α2-Adrenergic receptors, which are also involved in the pathogenesis of major depression and its treatment, also impacts the population of immature neurons in the PCX layer II (Vadodaria et al., 2017). Taking into account all these results, there is enough evidence to promote the study of the immature neurons in the layer II of the cerebral cortex of humans, specially comparing major depression patients with healthy controls.

In conclusion, our results strongly indicate a prominent role of D2r and PSA-NCAM, on the differentiation of immature neurons in the adult PCX, although further analyses closely following the final development of these cells should be necessary to understand this mechanism. Interestingly, together with previous evidence, our data also suggest that this neuronal cell population is the target of drugs affecting the monoaminergic systems and its involvement in the etiopathology of psychiatric disorders in which these systems are altered.

## Data Availability Statement

The datasets for this article are not publicly available. Requests to access the datasets should be directed to JN, nacher@uv.es.

## Ethics Statement

The animal study was reviewed and approved by the Committee on Bioethics of the University of Valencia.

## Author Contributions

SC, EC-G, and JN designed the experiments. SC, YG, and EC-G performed the experiments. SC and JN wrote the manuscript. JN supervised the experiments and wrote the final version of the manuscript. All authors contributed to the article and approved the submitted version.

## Conflict of Interest

The authors declare that the research was conducted in the absence of any commercial or financial relationships that could be construed as a potential conflict of interest.
